# Behavioral interfaces for executable DSLs

**DOI:** 10.1007/s10270-020-00798-2

**Published:** 2020-04-23

**Authors:** Dorian Leroy, Erwan Bousse, Manuel Wimmer, Tanja Mayerhofer, Benoit Combemale, Wieland Schwinger

**Affiliations:** 1 JKU Linz, Linz, Austria; 2 TU Wien, Vienna, Austria; 3 CDL-MINT, Linz, Austria; 4 UT2J, Toulouse, France

**Keywords:** Language engineering, Domain-specific language, Model execution

## Abstract

Executable domain-specific languages (DSLs) enable the execution of behavioral models. While an execution is mostly driven by the model content (e.g., control structures), many use cases require *interacting* with the running model, such as simulating scenarios in an automated or interactive way, or coupling the model with other models of the system or environment. The management of these interactions is usually hardcoded into the semantics of the DSL, which prevents its reuse for other DSLs and the provision of generic interaction-centric tools (e.g., event injector). In this paper, we propose a metalanguage for complementing the definition of executable DSLs with explicit behavioral interfaces to enable external tools to interact with executed models in a unified way. We implemented the proposed metalanguage in the GEMOC Studio and show how behavioral interfaces enable the realization of tools that are generic and thus usable for different executable DSLs.

## Introduction

A large amount of domain-specific languages (DSLs) are used to represent behavioral aspects of systems in the form of *behavioral models* (e.g., [4, 13, 16, 29, 31]). To enable the dynamic analysis of such models, a lot of efforts have been made to facilitate the design of so-called *executable DSLs* (xDSLs) (e.g., [3, 9, 12, 17, 24, 38, 42]), which enable the execution of conforming models. Two approaches are commonly used to define execution semantics, namely operational semantics (i.e., interpretation) and translational semantics (i.e., compilation). In this paper, we focus on operational semantics and more precisely on discrete-event operational semantics.

While the execution of a behavioral model is mostly driven by its content (e.g., conditionals, transitions, method calls), many cases require to *interact* with the running model [8]. For instance, simulating a specific execution scenario requires sending *stimuli* to the model (e.g., sending signals to a UML activity diagram) and checking whether the model reacts as expected. Likewise, coupling a behavioral model with models representing other parts of the system or its environment requires stimuli originating from these additional models. In addition, depending on the circumstances, it can be preferable to send stimuli to a model in an automated way (e.g., programmatically through a test runner) or in a manual way (e.g., using an event injector).

In order to interact with the execution of a model, the operational semantics (i.e., the interpreter) of the xDSL must fulfill at least two requirements. First, the semantics must define how conforming models may react to incoming stimuli, and when outgoing stimuli are emitted to external tools. This can include defining the possible *types of stimuli* (e.g., the notion of signal in UML) and defining the *handling logic* for each type of stimuli (e.g., what happens when a signal occurs). Second, some form of *messaging architecture* must be in place to enable the passing of stimuli to and from a running model. For instance, external tools can be allowed to modify some parts of the execution state of the running model or can trigger stimuli using some API exposed by the interpreter.

However, the form of both the stimuli and the handling logic may vary greatly from one DSL to another. Consequently, these requirements are in practice fulfilled on a language basis, each xDSL having its own set of tools interacting with running models (e.g., see Papyrus^1^ for UML and SysML). Yet, specifying in a unified way the interaction capabilities of xDSLs would yield several benefits. First, it would enable the definition of generic interaction-centric tools (e.g., a test runner, an event injector). Second, it opens the possibility of defining a hierarchy of more abstract events that can be implemented by similar DSLs, hence enabling the definition of generic tools for DSL families, or what we reference to as genericity through abstraction.

To unlock these benefits, we introduce a new metalanguage to complement the definition of xDSLs with *behavioral interfaces*. A behavioral interface defines a set of *events* specifying how external tools can interact with models that conform to xDSLs implementing the interface. Additionally, we define two types of relationships involving behavioral interfaces: the *implementation* and the *subtyping* relationships. An implementation relationship ties a behavioral interface to a given operational semantics implementation. Subtyping relationships allow to build event abstraction hierarchies, indicating that events from one interface can be abstracted or refined as events from another interface. Through subtyping relationships, a given behavioral interface can be subtyped by several xDSLs, enabling the reuse of tools specific to this interface across these DSLs. The semantics of the proposed metalanguage for behavioral interfaces is defined by a generic, yet configurable, *event manager*. At runtime, this event manager leverages the definition of subtyping and implementation relationships to translate event occurrences from external tools into actual behavior, and conversely reacts to observable behavior by emitting event occurrences to external tools. Tools can then automatically discover which behavioral interfaces are implemented by an xDSL—both through implementation and subtyping relationships—and thus how to interact with conforming models.

To evaluate our contribution, we fixed a list of requirements for the different aspects of the approach. To test whether the proposed approach fulfills these requirements, we implemented the behavioral interface metalanguage as part of the execution framework of the GEMOC Studio [5], an Eclipse-based language and modeling workbench for xDSLs. We evaluate the approach with three demonstration cases. In the first one, we show that the proposed metalanguage can be used to define the behavioral interface of two xDSLs. In the second one, we show that behavioral interfaces enable the definition of generic tools and their use across several xDSLs. In the third one, we show that a single behavioral interface can be subtyped by several xDSLs, allowing to interact with and reason about the execution of conforming models through a common behavioral interface.

The remainder of this paper is structured as follows. Section 2 presents the background and the motivation for this work, as well as the requirements for the approach. Section 3 provides an overview of our contributions. Section 4 presents the definition of behavioral interfaces and of implementation and subtyping relationships. Section 5 presents a possible strategy for realizing implementation and subtyping relationships, managing event occurrences and integrating the approach with metalanguages for defining the execution semantics of xDSLs. Section 6 presents the evaluation of the approach. Section 7 discusses the related work. Finally, a conclusion and future research directions are presented in Sect. 8.

## Background and motivation

In this section, we precisely scope the xDSLs considered in our approach and then motivate our approach using an illustrative example.

### Background on executable DSLs

An xDSL is composed of an abstract syntax defining the concepts of the considered domain and an execution semantics defining the meaning of these concepts. In this paper, we focus on DSLs where (1) the abstract syntax is provided as a *metamodel* defined using a metamodeling language (e.g., MOF [32] or Ecore [40]) and (2) the execution semantics is provided as an operational semantics (i.e., an interpreter).

In addition to the metamodel defining its abstract syntax, an xDSL can expose several structural language interfaces constituting its available model types [11, 15, 39]. These model types define a set of metaclasses and structural features that are guaranteed to be present in the metamodel constituting the abstract syntax of the DSL and thus supported by its conforming models.

The considered operational semantics are those composed of a data structure representing the *model state* and a set of *execution rules* altering this model state.

#### Definition 1

We define the operational semantics of an xDSL as a tuple $\langle DM , ER \rangle$ where $DM$ is its dynamic metamodel and $ER$ its set of execution rules.

The model state is defined in an *execution metamodel* extending the abstract syntax metamodel using a non-intrusive extension mechanism, such as *package merge* [30] or *aspect weaving* [20]. The execution rules perform an in-place endogenous transformation on this model state. This model transformation effectively results in the execution of the model and can be implemented by various means (e.g., programming languages, model transformation languages). The scheduling of the execution rules is defined by the language engineer and can be influenced by the metalanguage used to write them. For instance, it can be implicit, if the metalanguage works in a more declarative way (e.g., Henshin). Alternatively, it can be explicitly defined in the execution semantics, if execution rules are directly calling other execution rules, following an imperative programming style. While the operational semantics can handle time (e.g., through a central clock), the proposed approach is time-agnostic.

#### Definition 2

We define an xDSL as a tuple $\langle AS , OS \rangle$ where $AS$ is its abstract syntax and $OS$ its operational semantics.

**Fig. 1:**
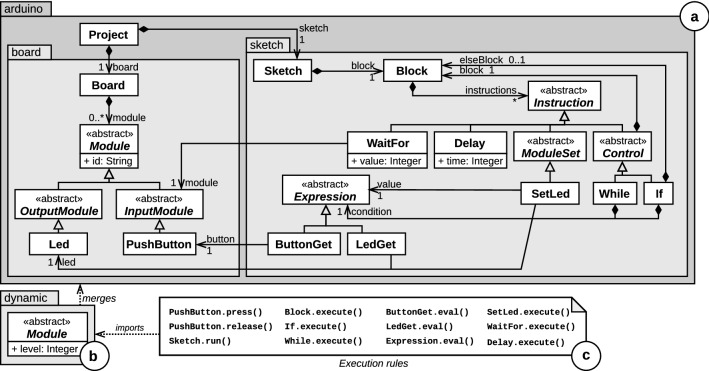
Arduino executable DSL definition

Figure 1 shows the definition of the Arduino xDSL, which will be used as a running example throughout this paper. The abstract syntax of the DSL is defined as a metamodel (**a** in Fig. 1). A Project contains a Board and a Sketch. The board of the project represents the physical Arduino board on which the sketch of the project is executed. A Board contains Modules, which have an id attribute. A Module can either be an OutputModule, such as a Led, or an InputModule, such as a PushButton. Being a program to be executed on a Board, a Sketch contains a Block of Instructions that can be Control, ModuleSet, Delay or WaitFor instructions. Control instructions come in the usual forms of If and While instructions. They contain a Block (or potentially two for the If instruction) and a condition in the form of an Expression. For the sake of brevity, the whole class diagram of Expression is not shown here, except for the ButtonGet and LedGet classes, which, respectively, point to a PushButton and a Led. The ModuleSet class is further specialized for each OutputModule: Here, SetLed is a ModuleSet instruction for Led modules, setting the level attribute of its associated Led to the result of the evaluation of its value expression. The Delay instruction suspends the execution for the specified amount of milliseconds. The WaitFor instruction points to an InputModule and suspends the execution until the level of the referenced module reaches the provided value.

The bottom part of Fig. 1 shows the two parts of the operational semantics of the Arduino DSL. The execution metamodel (**b** in Fig. 1) extends the Module class with the level integer attribute, indicating the logic level of the signal transiting between a Module and its containing Board. For Led modules, the level represents whether the LED is lit or not, whereas for PushButton modules, it indicates whether the button is currently pressed or not. The execution rules (**c** in Fig. 1) import this execution metamodel and consist of model transformations defining how the state of a running model is altered. In the case of the Arduino DSL, only the level attributes of Led and PushButton elements can be changed, either by the SetLed.execute rule for Led modules, or PushButton.press and PushButton.release for PushButton modules. As the execution semantics of the Arduino DSL is implemented as a visitor, the scheduling of the execution rules is determined internally. The Sketch.run rule is the entry point rule of the DSL: It starts the model transformation resulting in the execution of conforming models. To this end, it calls the Block.execute rule of its contained Block, thereby starting the visit of the containment tree of the running model. The Block.execute rule sequentially calls the execute rule of the instructions it contains. The If.execute rule calls the Block.execute rule on its block (resp. elseBlock), if its condition evaluates to true (resp. false). Similarly, the While.execute rule calls the Block.execute rule on its block, while its condition evaluates to true. The remaining execution rules are dedicated to the the implementation of the behavior of the SetLed instruction and of the waiting mechanism of the Delay and the WaitFor instructions. The complete definition of the DSL is available on Github.^2^

### Motivation and requirements

**Fig. 2:**
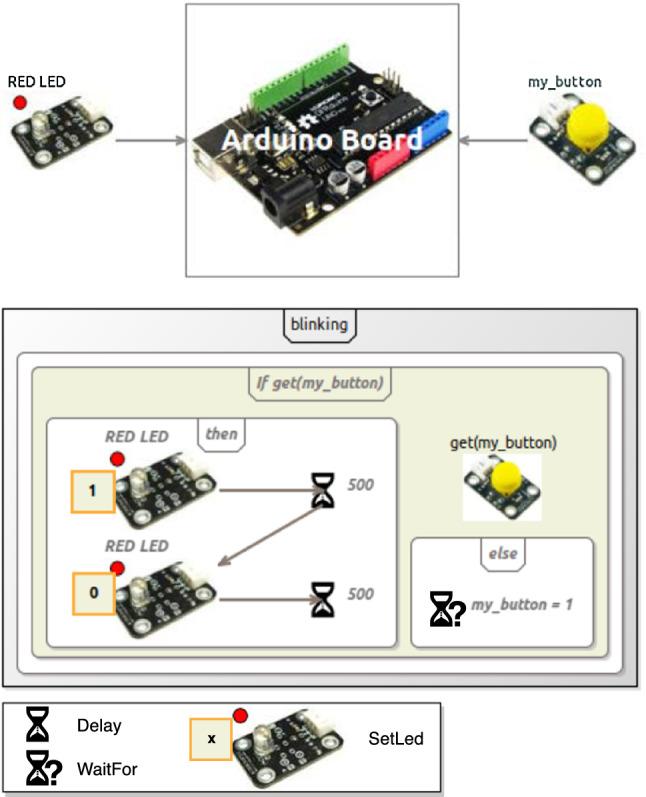
Example Arduino model

Figure 2 shows an example model conforming to the Arduino DSL. This model represents an Arduino circuit with one button and one LED, where the LED blinks while the button is pressed and remains off otherwise.

If we consider an execution of this model where the button is pressed in the initial state and remains pressed in all states, we observe that the LED blinks as defined in the model. However, such an execution scenario does not show whether the LED eventually stops blinking when the button is released, or more generally how the LED behaves with different scenarios of button pressings. To test more complex execution scenarios, the modeler must be able to change the state of the button *during* the execution of the model.

**Fig. 3:**
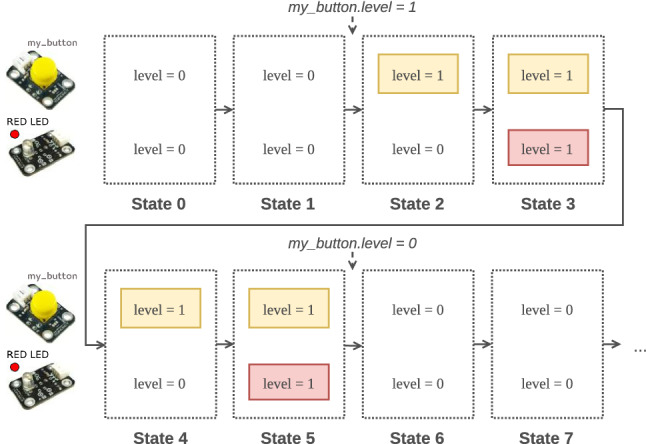
Execution of the Arduino model (Fig. 2) where the PushButton is only pressed between states 2 and 5

Since our operational semantics does not provide any explicit way to interact with a running model, one possibility is for the modeler to directly modify the state of the model during its execution, effectively resulting in a form of stimulus. Figure 3 shows an execution trace where two changes (*my_button.level = 1* and *my_button.level = 0*) are made during the execution, the first changing the level attribute of the PushButton to 1 and the second changing it to 0. The modeler can thus effectively observe that the LED not only blinks when the button is pressed, but also stops blinking when the button is released. While this solution does allow the execution of specific scenarios, it has several limitations, which can be divided into two categories.

The first category of limitations relates to the way the possible stimuli that can be sent to models are defined. Manipulating these stimuli as model changes is both a cumbersome process and error-prone process for modelers. For instance, issuing the model changes corresponding to a given stimuli and interpreting observed model changes both necessitate extensive knowledge of the operational semantics of the DSL, which modelers are not assumed to have. In addition, it can result in unsound behavior with regard to the operational semantics of the DSL. For example, the semantics of a language may restrict the subset of the execution state of a conforming model that can be affected by an external stimulus (e.g., the status of an InputModule of an Arduino board), while the remainder of the state should only be affected by the inner operational semantics (e.g., the status of an OutputModule). One way to circumvent these problems is to provide a clearly defined way for language engineers to define the *behavioral types* of xDSLs. The purpose of these behavioral types is to expose the domain-specific stimuli of an xDSL as first-class entities that are part of the language definition. A widely adopted approach for the reification of stimuli types is to consider such stimuli as occurrences of well-defined *events* (i.e., their type). This would allow language engineers to attribute a type to the stimuli received and sent by models and thus facilitate both their manipulation by modelers and *sound* interaction with models. For example, in Fig. 3, the two model changes made by the stimuli during the execution correspond to a particular button being pressed and released. From the perspective of the modeler, this is not strikingly clear. The language engineer can improve this by defining two types for these stimuli: the *pressed* and *released* stimuli types, which both convey more domain semantics than low-level model changes. From these limitations, we can define a first requirement for the approach as follows:

**Req. 1**: “Provide an explicit and unified way to *define* the behavioral type of an xDSL, i.e., how to soundly interact with any model conforming to the DSL.”

The second category of limitations relates to the way stimuli are sent to and received from the running model and to the soundness of the resulting behavior. For example, the operational semantics may only allow some stimuli to affect the execution state at certain points in time or when it is in a specific state. Since arbitrary transformations may break these constraints, it appears important to control which and when changes of the model state are allowed, e.g., by only allowing specific execution rules to be called in reaction to stimuli and under specific circumstances. Another limitation is related to the concurrent execution of multiple transformations on the same model state, which can quickly lead to undefined behaviors when some values are simultaneously changed externally and by the operational semantics. It appears therefore important to also control when stimuli-triggered changes can be applied to the model state, e.g., by delaying their application until the currently executing rule yields back control if it is a run-to-completion rule call. Moreover, xDSLs are only as useful as the richness of the ecosystem of tools defined for them. One limitation of the solution proposed in Fig. 3 is that it does not provide a unified way to send stimuli to the model. This hampers the definition of tools interacting with running models. Conversely, this solution does not provide a clear way for the model to emit stimuli of its own toward external tools. Both cases thus require ad hoc techniques to either inspect observable parts of the state of a running model (e.g., to detect when a LED is switched on or off), or send stimuli to running models. From these limitations, we can define a second requirement on the approach as follows:

**Req. 2**: “Provide a unified way to *interact* soundly with models conforming to xDSLs implementing one or several behavioral types.”

However, in model-driven engineering, parts of a system can be modeled using various DSLs fitting different needs such as model checking, simulation, animation and so on. Due to their individual particularities, such DSLs potentially accept and expose different events. This prevents modelers from using the same events to interact with models conforming to different DSLs despite representing the same part of the system. One way to answer this problem is to allow language engineers to define a set of events abstracting various events defined for each of these DSLs. From there, language engineers can define how this set of abstract events maps to other sets of events, effectively defining overlapping event abstraction hierarchies. Using these mappings at runtime to translate event occurrences would enable modelers to interact with models conforming to any of the covered DSLs through this single set of abstract events. In addition, language engineers would foster the emergence of families of xDSLs supporting a shared set of abstract events. For example, the Arduino model shown in Fig. 2 could be the realization of a specification available as a model conforming to a State Machine DSL. By defining how events supported by State Machine models can be mapped to events supported by Arduino models, a language engineer would enable interaction with both kinds of models using the same set of events. From this scenario, a third and last requirement on the approach can be formulated:

**Req. 3**: “Support the definition of overlapping event abstraction hierarchies for xDSLs.”

To support these scenarios, we propose a new metalanguage to specify in a unified way the behavioral types of xDSLs under the form of *behavioral interfaces*, thereby fulfilling **Req. 1**. Implementation relationships can then be established between xDSLs and their implemented behavioral interfaces. At runtime, these relationships configure a generic event manager to enable safe interaction with the running model, while keeping a clear separation between the implementation of an xDSL and its interfaces. In turn, this event manager exposes the available behavioral interfaces of the running model, thereby enabling the definition of generic interaction-centric tools and fulfilling **Req. 2**. Finally, we introduce subtyping relationships between behavioral interfaces, allowing to define event abstraction hierarchies and thus fulfill **Req. 3**. We provide an overview of the complete approach in the following section.

## Approach overview

We provide in this section an overview on both the design of the approach and its envisioned use by developers.

### Design overview

**Fig. 4:**
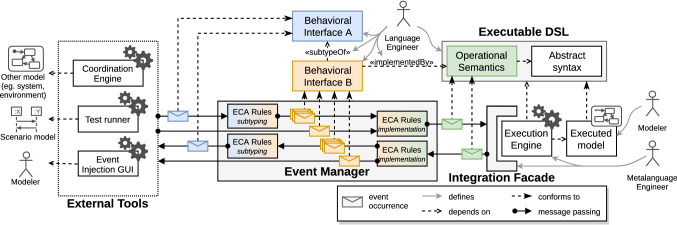
Overview of the approach

Figure 4 depicts an overview of the proposed approach. On the top right corner, the xDSL complies with the definition given in Sect. 2.1. As such, it contains an abstract syntax as a metamodel and an operational semantics with both a set of execution rules and a data structure defining the model state. On the bottom right corner is shown a running model whose static content conforms to the abstract syntax and whose dynamic state conforms to the execution metamodel. Next to it, the *execution engine* is able both to apply any execution rule of the operational semantics on the running model and to notify execution observers when execution rules are applied. Such an execution engine is based on our previous work on decoupling operational semantics from execution observers [5, 6].

On the left, examples of external tools that require interacting with the running model are represented.

- A coordination engine managing the communication with other models—representing some part of the environment or other parts of the system—during an execution.
- A test runner executing a specific scenario model, alternating between sending sequences of stimuli to the model and checking whether a proper sequence of stimuli are received from the model in return.
- An event injection GUI complementing the classic stepping operators of interactive debuggers (e.g., step into, step over) with the capability to manually send domain-specific stimuli to the running model, and to observe the stimuli produced in reaction.

For language engineers, developing such tools as needed for each new xDSL is both a tedious process and error-prone process. Providing to language engineers a unified way to define the possible interactions with models conforming to any xDSL would allow them to define generic tools instead. To achieve this for any xDSL included in our scope (see Sect. 2), we introduce *behavioral interfaces* as behavioral types of xDSLs.

Using the proposed approach, language engineers can define behavioral interfaces, or reuse existing ones, to type their xDSLs based on the runtime interaction capabilities offered by their conforming models. When external tools discover the behavioral interfaces of an xDSL, they are informed of the kind and form of stimuli that can be emitted and/or received by conforming models. This then allows modelers and other models to interact with conforming models through these tools.

Such an interface consists of an *event metamodel* that defines the exact set of *events* that are relevant to the interface purpose and/or domain. First, this means defining the events whose occurrences can be *accepted* or *exposed* by models conforming to xDSLs typed by the behavioral interface. Second, this means specifying the nature and structure of the data that can potentially be carried out by occurrences of these events.

As shown in Fig. 4 by the dependency between behavioral interface B and the operational semantics, language engineers can type their xDSL by a given behavioral interface by providing an *implementation relationship* between the interface and the xDSL, which amounts to nominal typing [36]. This implementation relationship describes how the xDSL provides the interaction capabilities that are expected a language typed by the behavioral interface. In practice, this is done by detailing how occurrences of the events defined in the interface translate in terms of actual behavior, and vice-versa. As a supplementary contribution, we provide a systematic way to generate the behavioral interface implemented by an xDSL, as well as the implementation relationship between them.

In addition, we introduce nominal subtyping [36] for behavioral interfaces in the form of *subtyping relationships*, illustrated between behavioral interfaces A and B in Fig. 4. By defining a subtyping relationship between two behavioral interfaces, language engineers designate one interface as the subtype and the other as the supertype. A subtyping relationship then dictates what patterns of accepted (resp. exposed) event occurrences from the supertype (resp. subtype) translate to which event occurrences from the subtype (resp. supertype), in what order and carrying what data. Using subtyping relationships, language engineers can capitalize on the behavioral similarities of different xDSLs to define tools that are both specific to these similarities and reusable across any DSL exhibiting them.

Both implementation and subtyping relationships are realized through Event-Condition-Action (ECA) rules, as shown in Fig. 4. These rules are triggered when a pattern of event occurrences—the event part of the rule—from one side of the relationship is observed, given that their associated condition (e.g., an OCL query, a Java predicate) is satisfied. When triggered, their action part translates the observed event occurrences into new event occurrences belonging to the other side of the relationship. As shown in the figure, ECA rules are managed by the event manager, a component able to route event occurrences from and to various ECA rules, the execution engine and the external tools.

Finally, we propose an integration facade for the event manager that acts as an intermediary between the execution engine and the aforementioned event manager. While this integration facade is specific to the metalanguage that is used to define the operational semantics of xDSLs, the rest of the approach is agnostic to any such metalanguage. Furthermore, defining this facade for a metalanguage enables the approach for any xDSL whose operational semantics is defined with this metalanguage.

In the end, the three main constituents of the approach—the behavioral interface metamodel, the relationships and the event manager—form a metalanguage to extend an xDSL with an event handling component. The behavioral interface metamodel is used to define the abstract syntax of such language extensions, while the relationships define their operational semantics. Models conforming to this language extension are occurrences of events defined in behavioral interfaces. At runtime, the event manager acts as the engine executing the operational semantics of the language extension (i.e., manage event occurrences and relationships), thereby forming an interpreter for the language extension.

### Usage overview

In the scope of the paper, we distinguish three kinds of users for the approach: metalanguage engineers, language engineers and modelers. We describe hereafter each of these kinds, which are represented in Fig. 4.

Metalanguage engineers are the users that design metalanguages or adapt existing languages to be used as metalanguages to define xDSLs. In addition, they develop the environment necessary to execute models, which we designate as execution engine, and possibly tooling that is generic to any language designed with their metalanguage (e.g., debugger, tracing facilities). With the proposed approach, they can provide an integration facade for their execution engine to enable any xDSL based on their metalanguage and implementing a behavioral interface to use existing generic interactive tools that work with any behavioral interface. This has a cost for metalanguage engineers, but we believe that, as their role is to provide facilities to create new languages, they have a great incentive to add such a facade. However, it is possible that in some cases the person with the role of language engineer can temporarily take on the role of metalanguage engineer to provide the integration facade through a pull request or a similar process. But in that case, the correctness of the integration facade still has to be assessed by a metalanguage engineer.

Language engineers are the users that design xDSLs using a metalanguage to define their execution semantics. These users also develop domain-specific tools for their languages, either from scratch or by reusing and/or extending generic tools provided by metalanguage engineers. With the proposed approach, they can provide or reuse behavioral interfaces as well as implementation and subtyping relationships between these interfaces and their xDSLs. In addition, language engineers can provide or reuse interactive tools that are specific to the behavioral interfaces implemented by their xDSLs. To enable this, they need to learn the proposed metalanguage and how to define relationships, but we believe that the benefits outweigh the costs as soon as this enables the direct reuse of even a small set of tools.

Lastly, modelers are the users designing models conforming to xDSLs. Depending on how supported a given xDSL and the metalanguage used to define its execution semantics are, modelers will have access to a number of tools to aid them in their endeavor. With the proposed approach, modelers get access to any generic interactive tooling, as well as any tooling specific to a behavioral interface implemented, either directly or transitively, for the xDSLs they use.

In what follows, we first provide a specification for behavioral interfaces, and implementation and subtyping relationships in Sect. 4. Then, we detail one possible strategy to realize the proposed approach in Sect. 5.

## Behavioral interface and relationships

In this section, we first specify what are behavioral interfaces in Sect. 4.1, then we give a specification of implementation and subtyping relationships in Sect. 4.2.

### Behavioral interface

In this subsection, we introduce the notion of *behavioral interface* for xDSLs. Behavioral interfaces declare the set of domain-specific stimuli that can be send to or received from models conforming to an implementing xDSL.

#### Behavioral interface metamodel

**Fig. 5:**
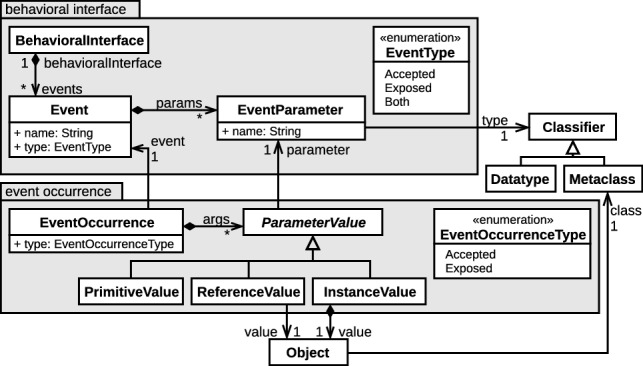
Behavioral interface metamodel

Figure 5 shows a metamodel formalizing the minimal set of syntactical elements required to define behavioral interfaces and occurrences of the events declared therein and instantiated at runtime. A behavioral interface is composed of Event elements defining the possible interactions with models conforming to xDSLs typed by the interface. Events have a name and can either be accepted events, exposed events or both, as indicated by their type. Events also have a set of EventParameters that define the data carried out by their occurrences. A parameter is identified with a name and can either carry primitive values or objects values, as determined by its type. Primitive values are typed by a DataType, and object values are typed by a Metaclass. Metaclasses referenced as the type of an event parameter can belong to a specific domain, typing the interface to that domain, or be defined specifically for the behavioral interface (e.g., to carry complex data while keeping the interface self-contained). This allows a behavioral interface to be tied to a specific domain or to be as generic as desired.

To use a behavioral interface, it must be defined as a type for an xDSL, through an implementation relationship between the interface and the xDSL. Alternatively, this can be achieved through a subtyping relationship toward a behavioral interface that is defined as a type for the xDSL. Section 4.2 provides more details about implementation and subtyping relationships.

Once this is done, any tool working with xDSLs implementing either any behavioral interface or specific ones can be used with models conforming to the implementing DSL. Such tools send or receive instantiated events from the implemented interfaces under the form of EventOccurrence elements. These elements have an event reference to the Event of which they are an occurrence. They also have a type attribute indicating whether they are accepted or exposed event occurrences. Additionally, event occurrences contain the values attributed to each parameter of their Event as ParameterValue elements. According to the type of their corresponding parameter, these parameter values can be PrimitiveValue elements (not detailed in Fig. 5) or object values. In the case of object values, we make a distinction between references to objects contained elsewhere (ReferenceValues) and objects that are directly contained by the event occurrence (InstanceValues). This allows to pass references to elements of the running model as parameters, as well as objects created for the sole purpose of sending the event occurrence. The referenced model elements are accessed through the read-only structural language interface of the metamodel they conform to, thereby preventing their unauthorized modification. In addition, this allows for event parameters to reference metaclasses that are compatible with several xDSLs, if these references are typed by metaclasses contained in a model type common to these DSLs. In the proposed approach, event occurrences do not need to be contained in another entity. However, a language engineer aiming to provide tooling that revolves around event occurrences can define metamodels (e.g., scenario or trace metamodels) with a composition relationship toward event occurrences, which is the approach adopted in the GEMOC Studio.

##### Definition 3

Let *I* be a behavioral interface. $$ Occ _I$$ denotes the set of all the event occurrences that can be instantiated from the events defined in *I*.

For instance, in the Arduino DSL, an event signaling the push of a button will carry a reference (thus a ReferenceValue) to the button being pushed, whereas in UML State Machines, an equivalent event would carry an instance of UML event only created to send the event occurrence (thus an InstanceValue), named "button_pushed" and itself carrying the identifier of the button being pushed.

#### Examples of behavioral interfaces

**Fig. 6:**
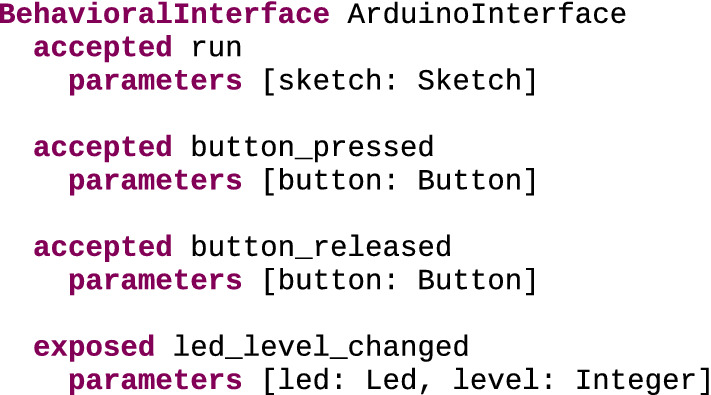
Behavioral interface for Arduino DSL

ArduinoInterface Figure 6 shows a possible behavioral interface for the Arduino DSL, using the textual concrete syntax of the behavioral interface metalanguage. This interface defines three accepted events and one exposed event. As they are accepted events, occurrences of run, button_pressed and button_released can be sent by external tools, triggering specific behavior in executed models. Occurrences of run are meant to start the execution of the Sketch element provided for the sketch argument. In the case of button_pressed and button_released, occurrences thereof are meant to change the state of the provided Button element to pressed or released. Conversely, occurrences of the exposed event led_level_changed can be emitted and exposed to external tools when specific behaviors are detected in executed models. These occurrences carry two parameters: a Led element and the new value of its pinValue attribute.

**Fig. 7:**
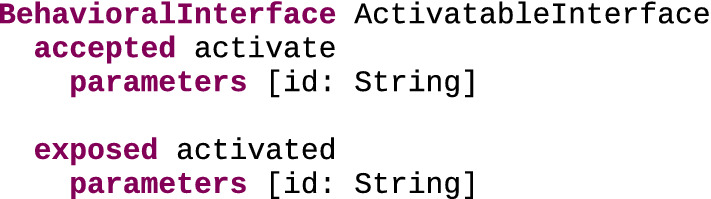
ActivatableInterface behavioral interface

ActivatableInterface Figure 7 shows a behavioral interface meant for xDSLs whose conforming models contain elements that can be activated, which we refer to as the ActivatableInterface. This language interface can be implemented by xDSLs to extend their definition with the handling of two events relating to the activation of elements: activate, which is an accepted event meant to trigger the activation of an element, and activated, which is an exposed event notifying that an element has been activated. Both events have an idString parameter identifying which element is affected by the event. This makes the interface quite generic and thus usable by various xDSLs, as long as the provided String parameters allow to identify elements of interest.

#### Behavioral interface generation

**Fig. 8:**
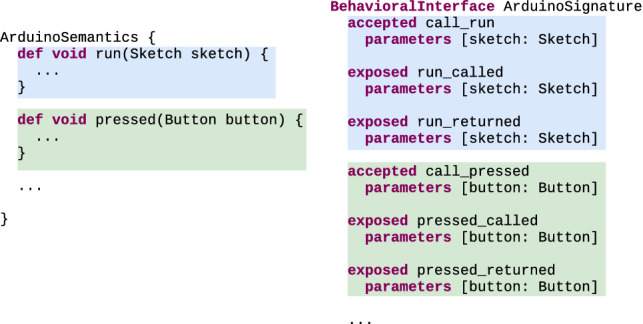
Excerpt of behavioral interface (right) derived from the Arduino DSL operational semantics (left)

Figure 8 shows on the right an excerpt of the most precise behavioral interface (here called ArduinoSignature) that can be defined for the Arduino DSL and, on the left, how it maps to its operational semantics.

For each execution rule of the operational semantics, the behavioral interface contains (1) an accepted event triggering calls to the execution rule, (2) an exposed event signaling the start of the execution of the rule and (3) an exposed event signaling the end of the execution of the rule. Any naming scheme can be used to uniquely name these events. In our case, we chose to append or prepend "called", "returned" or "call" to the name of the execution rule. The parameters of these events are identical to the parameters of their corresponding execution rule.

To streamline the application of the approach to xDSLs, we implemented a generator that systematically derives a behavioral interface to serve as the most precise behavioral type of an xDSL. The generator performs a static analysis of the code of the operational semantics of the DSL to extract all the execution rules it contains. The generator then generates the most precise interface of the DSL based on the signatures of the execution rules, in accordance with the specification provided above. This generator also provides a corresponding trivial implementation relationship, mapping directly each generated event to its associated execution rule. To complement the provision of such a generator, metalanguage engineers can supply an annotation system or a similar mechanism that allows language engineers to annotate which execution rules will result in accepted and/or exposed events.

### Implementation and subtyping relationships

In order to use behavioral interfaces as types for xDSLs, it is necessary to define both what is an *implementation relationship* between a behavioral interface and an xDSL and what is a *subtyping relationship* between two behavioral interfaces. This subsection first lays some preliminary definitions related to operational semantics and events and then specifies both kinds of relationships.

#### Preliminary definitions

We hereafter introduce the concepts that will be used to specify implementation and subtyping relationships.

*Operational semantics* The proposed approach relies on the translation of accepted event occurrences into actual behavior—e.g., calls to execution rules of the operational semantics—and conversely on the translation of behavior into exposed event occurrences. Essentially, for this approach, interactions with the operational semantics can be considered of two kinds: *call requests* and *call notifications*.

Call requests can be issued to request the execution of a specific execution rule of the operational semantics. Such requests must supply the name of the execution rule to be called as well as the arguments to be passed when the call is eventually carried out. Additionally, in some cases it may be required to declare that a requested call must be performed in a run-to-completion way, meaning that no other call request should be handled as long as the run-to-completion one has not returned. For example, call requests to the PushButton.press and PushButton.release execution rules of the Arduino DSL should be handled in a run-to-completion way, as calls to these rules model an instantaneous behavior during which nothing else can happen. For this reason, call requests can individually be configured to be handled in a run-to-completion way.

Conversely, call notifications carry a return Boolean indicating whether a particular execution rule has been or is about to be executed. Call notifications supply the name of the execution rule that has been or is about to be executed, as well as the arguments passed to the execution rule at the moment of the call. Additionally, if the notification informs that an execution rule has been fully executed, it also carries the resulting value of the call, if applicable, as well.

There are numerous ways to define how call requests can be handled by the operational semantics as well as how call notifications are emitted. How this is done depends heavily on the metalanguage used to define the operational semantics of xDSLs. For instance, if the metalanguage being used is a graph transformation language like Henshin [2], call requests will likely be handled in between the application of transformation rules, as the model state in the middle of the application of a rule is not consistent. Therefore, we do not restrict our approach to any strategy, but propose one such strategy compatible with our technological space of reference (i.e., where the operational semantics is written in an object-oriented programming language and its execution orchestrated by an execution engine) in Sect. 5.2.

*Event stream* We consider that event occurrences, call requests and call notifications are observed from and inserted into an ordered event stream. This event stream can then be projected on each behavioral interface acting as behavioral type for an xDSL.

*Event-Condition-Action rules* We rely on Event-Condition-Action (ECA) rules to define implementation and subtyping relationships. ECA rules consist of three parts: an *event part* which specifies which stimuli triggers the rule, a *condition part* which is a predicate that must evaluate to true for the action to be executed and an *action part* which consists of a behavior to execute when the rule is triggered and its condition satisfied.

A wide range of valid strategies exist to define ECA rules, which mostly depend on how the stream of event occurrences is modeled. Therefore, the approach is not restricted to a particular strategy, but one such strategy is proposed in Sect. 5.1.

*Event abstraction hierarchy* Event abstraction hierarchies consist of layers of abstraction containing complex events defined over the layer below that provide a more detailed or refined view of the event stream.

*Pattern* One does not necessarily have control over the definition of the behavioral interfaces involved in a subtyping or implementation relationship. Thus, it follows that a one-to-one mapping cannot always be established between the events of two behavioral interfaces. (The same applies to events and call requests and notifications.) Therefore, to compensate for this potential discrepancy, a means to detect patterns of event occurrences or call notifications is required.

In this paper, we consider the definition of temporal pattern matching over a stream of occurrences of events from a behavioral interface and notifications of calls of execution rules from the operational semantics. Such patterns can be as simple as a single event occurrence, or be more complex like a sequence of several event occurrences in a particular order. Being able to specify and detect temporal patterns in turn enables the definition of ECA rules with a non-trivial event part.

#### Implementation relationship

A behavioral interface is said to be *implemented* by an xDSL when an *implementation relationship* is defined between the DSL and the interface. Intuitively, an implementation relationship between an xDSL and a behavioral interface guarantees that an observable behavior from the point of view of the interface can always be defined for every model conforming to this DSL. This means that the implementation relationship translates the internal behavior of models, defined with execution rule calls, into observable behavior, defined with occurrences of events of the implemented interface.

**Fig. 9:**
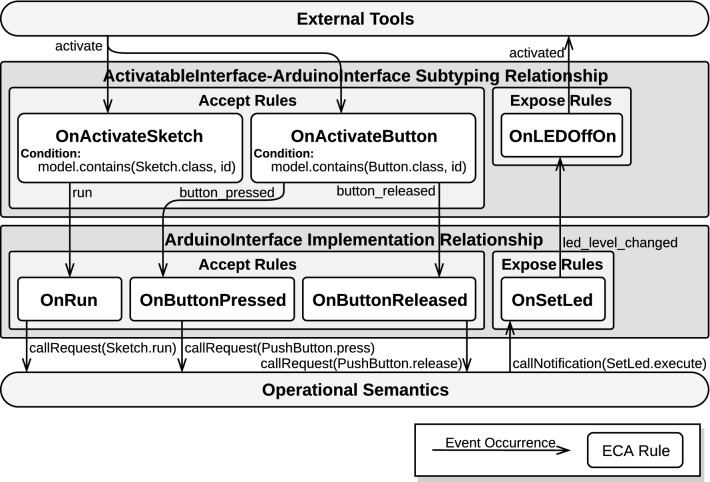
Relationships between the Arduino DSL and behavioral interfaces from Figs. 6 and 7

To provide a formal definition of implementation relationships, we rely on labeled transition systems (LTSs) to define the behavior of models and the behavior observable through an interface. We define LTSs as follows:

##### Definition 4

(*Labeled transition system*) An LTS is a tuple $\langle S, L, T \rangle$ where *S* is a set of states, *L* a set of labels and *T* a set of labeled transitions such that $T \subseteq S \times (L \cup \lbrace \tau \rbrace ) \times S$.

In addition, we introduce the following notations:

- $p \xrightarrow {\lambda } q$ denotes that there is a transition between *p* and *q* which is labeled $\lambda$,
- $$p~\xrightarrow {\lambda _1 \cdot \cdots \cdot \lambda _n}~q$$ denotes that $$p~\xrightarrow {\lambda _1}~\ldots ~\xrightarrow {\lambda _n}~q$$,
- $p \rightarrow {\lambda } q$ denotes that there is an arbitrary number of transitions labeled $\tau$ and a transition labeled $\lambda$ such that $p \xrightarrow {\tau } \ldots \xrightarrow {\lambda } \ldots \xrightarrow {\tau } q$,
- $$p~\rightarrow {\lambda _1 \cdot \cdots \cdot \lambda _n}~q$$ denotes that $$p~\rightarrow {\lambda _1}~\ldots ~\rightarrow {\lambda _n}~q$$,
- given a set of LTSs $$ LTS $$, $$ States ( LTS )$$ denotes the union set of all the states of the LTSs in $$ LTS $$,
- given a behavioral interface *I*, $$ LTS _I$$ denotes the set of all the LTSs that can be defined using a subset of $$ Occ _I$$ as their set of labels.

In our formal definitions, we abstract discrete-event models as LTSs follows;

- The set of states is defined as the set of possible dynamic states of the model,
- The set of labels is defined as the set of all possible calls that can be performed on the subset of execution rules of the operational semantics exposed by the language engineer,
- The set of transitions is defined according to the possible transitions between these states. Transitions that do not involve a call to an execution rule exposed by the language engineer are labeled with $\tau$.

Next, to formally express the behavioral equivalence between the internal behavior of a model and its observable behavior through an interface, we introduce weak and strong parameterized simulation, a variant from weak and strong simulation introduced by Milner in [28], as follows.

##### Definition 5

(*Weak/strong parameterized simulation*) Let $$L_1$$, $$L_2$$ be sets of labels. Let $$ LTS _1, LTS _2$$ be the sets of LTSs that can be defined from $$L_1$$ and $$L_2$$, respectively. Let $${\mathcal {S}} \subseteq States ( LTS _1) \times States ( LTS _2)$$ be a binary relation. Let $$f{:}\,L_1 \times States ( LTS _2) \rightarrow ({\mathbb {N}} \rightarrow L_2)$$ be a function associating, to a label from $$L_1$$ and a state from $$ LTS _2$$, a sequence of labels from $$L_2$$. Then, $${\mathcal {S}}$$ is said to be a weak (resp. strong) simulation *parameterized byf* if, whenever $$p {\mathcal {S}} q$$, if $$p~\xrightarrow {\lambda }~p'$$, then there exists $$q'$$ such that $$q~\rightarrow {f(\lambda , q)}~q'$$ (resp. $$q~\xrightarrow {f(\lambda , q)}~q'$$) and $$p' {\mathcal {S}} q'$$. Given two LTSs *P* and *Q*, we say that *P* weakly (resp. strongly) simulates *Q* through *f* if there exists a weak (resp. strong) simulation parameterized by *f* from all states of *P* to states of *Q*.

Based on this definition, and more precisely on the definition for weak parameterized simulation, we can formally define implementation relationships as follows.

##### Definition 6

(*Implementation relationship*) Let $L = \langle AS , \langle DM , ER \rangle \rangle$ be an xDSL and *I* a behavioral interface. Let $DS$ be the set of all model states conforming to $DM$, and $RC$ the set of all execution rule calls that can be issued from $ER$. We say that *LimplementsI* if there exists a function $Implem {:}\, Occ _I \times DS \rightarrow ({\mathbb {N}} \rightarrow RC )$ such that, for every model *m* conforming to $AS$, there exists an observable behavior $b \in LTS _I$ such that *mweakly simulatesbthrough*$Implem$.

In practice, the $Implem$ function of an implementation relationship between a behavioral interface and a DSL constitutes a two-layer event abstraction hierarchy, realized through two sets of ECA rules, namely the accept rules and the expose rules. Broadly, accept rules define how event occurrences of the interface are translated into behavior, while expose rules define how behavior results in event occurrences being emitted.

Accept rules define both which event occurrences conforming to the behavioral interface trigger behavior in the executed model and which parts of the operational semantics of the corresponding xDSL are used for that purpose. Accordingly, the three parts of an accept ECA rule are defined as follows:

- *event*: an accepted event occurrence conforming to the implemented behavioral interface.
- *condition*: a predicate to be applied on the event occurrence and on the model state.
- *action*: specifies, as (possibly run-to-completion) call requests, which (possibly concurrent) sequence of execution rule calls of the DSL semantics must be requested, and with what parameters.

Conversely, expose rules define both which behaviors in the executed model result in event occurrences and which event occurrences are instantiated thereupon. Each expose ECA rule is structured as follows:

- *event*: a (possibly temporal) pattern to be matched over a stream of call notifications.
- *condition*: a predicate to be applied on the matching set of execution rule calls and on the model state.
- *action*: specifies which exposed event occurrence of the implemented interface must be emitted in response to the detected behavior.

The lower part of Fig. 9 shows an example of implementation relationship between the ArduinoInterface interface and our Arduino DSL running example. This relationship defines three accept rules and one expose rules. In this case, the ECA rules directly map occurrences of each event to a matching execution rule. For instance, occurrences of button_pressed are mapped to a call request for the PushButton.press execution rule and call notifications for the SetLed.execute execution rule are mapped to occurrences of led_level_changed. Note that more complex mappings could be included, such as a mapping instantiating two call requests in response to an event occurrence.

#### Subtyping relationship

A behavioral interface is said to be a *subtype* of another behavioral interface when a *subtyping relationship* is defined between them. Intuitively, a subtyping relationship between two behavioral interfaces guarantees that an observable behavior from the point of view of the supertype interface can always be defined for every observable behavior from the point of view of the subtype interface. This means that the subtyping relationship translates the behavior of models observed through the subtype interface into observable behavior defined with occurrences of events from the supertype interface. We formally define subtyping relationships using strong parameterized simulation as follows.

##### Definition 7

(*Subtyping relationship*) Let $I_1, I_2$ be two behavioral interfaces. Let $L = \langle AS , \langle DM , ER \rangle \rangle$ be an xDSL implementing $I_1$, and let $DS$ be the set of all model states conforming to $DM$. We say that $I_1$ is a subtype of $I_2$ if there exists a function $Subtype {:}\, Occ _{I_2} \times DS \rightarrow ({\mathbb {N}} \rightarrow Occ _{I_1})$ such that, for every model *m* conforming to $AS$ with observable behavior $b_1 \in LTS _{I_1}$, there exists an observable behavior $b_2 \in LTS _{I_2}$ such that $b_1$*strongly simulates*$b_2$*throughSubtype*.

In practice, the $Subtype$ function of a subtyping relationship is realized similarly to the $Implem$ function of implementation relationships: It constitutes a two-layer event abstraction hierarchy realized with a set of accept ECA rules and a set of expose ECA rules. Subtyping relationships between two behavioral interfaces designate one interface as the *supertype* and the other as the *subtype*. Accept (resp. expose) rules are tasked with translating accepted (resp. exposed) event occurrences of the supertype (resp. subtype) into accepted (resp. exposed) event occurrences of the subtype (resp. supertype). Accept rules are structured as follows:

- *event*: an occurrence of an accepted event from the supertype.
- *condition*: a predicate to be applied on the event occurrence and on the model state.
- *action*: specifies into which sequence of which accepted event occurrences of the subtype the event occurrence is translated.

Expose rules are structured as follows:

- *event*: a (possibly temporal) pattern to be matched over a stream of occurrences of exposed events from the subtype.
- *condition*: a predicate to be applied on the matching set of event occurrences and on the model under execution.
- *action*: specifies into which exposed event occurrence of the supertype the event pattern is translated.

The upper part of Fig. 9 shows a subtyping relationship between the ActivatableInterface interface as a supertype and the ArduinoInterface as a subtype. Through this relationship, the language engineer defines the mapping between activate and activated events and the run, button_pressed, button_released and led_level_changed events. In this example relationship, when a button is activated, it means it has been pressed and then released. Likewise, a LED is considered as having been activated if it has been switched from off to on. Two accept rules and one expose rule are defined as part of this relationship. The OnActivateSketch rule is triggered by occurrences of activate and has a condition stating that a Sketch element with a name corresponding to the id carried out by the event occurrence must exist in the running model. The OnActivateButton rule is also triggered by occurrences of activate but has a different condition, stating that a Button element with the appropriate name must exist in the running model instead. When triggered, the OnActivateSketch rule emits an occurrence of the run event, whereas the OnActivateButton rule emits two event occurrences: an occurrence of button_pressed and an occurrence of button_released. The OnLEDOffOn rule illustrates that ECA rules can be triggered upon the detection of a pattern of several event occurrences: Here, the rule is triggered when a pattern involving several occurrences of the led_level_changed is observed. When triggered, this rule directly instantiates an occurrence of activated, as it does not have a condition (or rather, its condition always returns true).

#### Discussion on substitutability

Defining implementation and subtyping relationships according to Definitions 6 and 7 guarantees that every model conforming to an implementing DSL has an observable behavior from the point of view of each implemented behavioral interface. This is, however, not sufficient to guarantee that, if two xDSLs implement the same behavioral interface, *every* model conforming to one DSL can be substituted with at least one model conforming to the other without any observable difference from the point of view of the implemented interface.

In other words, the fact that an xDSL implements a behavioral interface (either directly or transitively) does not mean that *any* behavior that can be specified with that behavioral interface can be observed from a model conforming to this DSL. An implementation or subtyping relationship achieving this would fulfill a DSL-equivalent of the Liskov substitution principle, as modelers would always be able to use another language implementing the same interface to define a model that can be substituted with a given model.

Proving that an xDSL has such implementation and subtyping relationships defined for a set of behavioral interfaces can be done with a static analysis, provided the xDSL has a formally defined operational semantics.

## Event management and metalanguage integration

Three points remain open in the definition of implementation and subtyping relationships:

1. how to define the ECA rules of a relationship,
2. how relationships deal with (1) receiving event occurrences/call notifications, (2) pattern matching of event occurrences and (3) instantiating and forwarding new event occurrences/call requests , namely the event management strategy, and
3. how call requests and notifications are linked to a given operational semantics implementation, namely the metalanguage integration strategy.

In this section, we first propose a possible strategy for event management (in Sect. 5.1), tackling points 1 and 2, and then detail one possible strategy for metalanguage integration (in Sect. 5.2), tackling point 3.

### CEP-based event management

Implementation and subtyping relationships, as introduced in Sect. 4.2, require a concrete strategy to define and manage enclosed ECA rules. In this section, we present a strategy based on complex event processing (CEP) and more specifically on Esper’s event processing language (EPL). First, we mention the salient features of CEP that make it an interesting candidate for event management in our approach. Then, we introduce the event manager component acting as an ECA rule engine. Next, we detail how event occurrences are modeled in Esper, and finally, we explain the design process of relationships and their ECA rules.

**Fig. 10:**
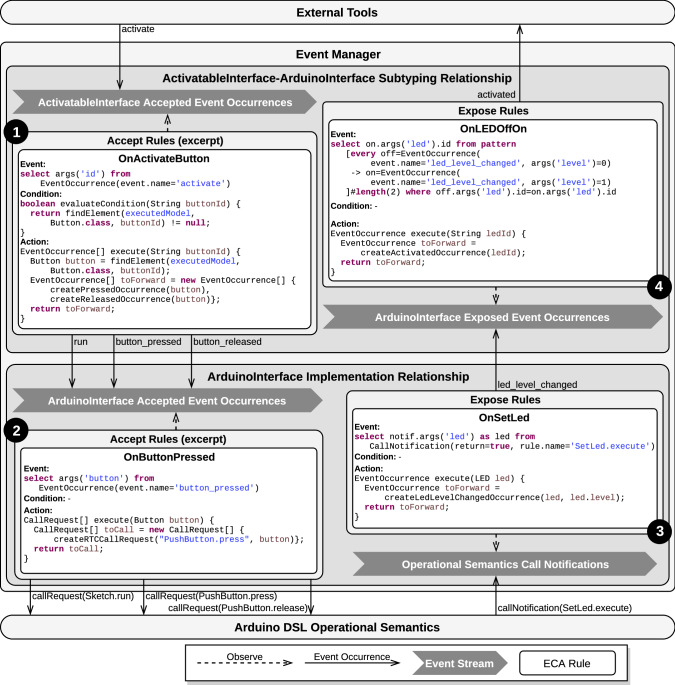
Excerpt of the CEP-based architecture applied to the Arduino DSL

#### Complex event processing

The goal of CEP is to identify meaningful events over streams of simpler events with queries on both the data carried out by the events and the before and after relationships between them. Essentially, CEP systems allow to perform temporal pattern matching over streams of events and produce a new stream of potentially overlapping complex events as a result. In this aspect, CEP is a paradigm that fits particularly well for the definition of event abstraction hierarchies [23], which are central to subtyping relationships between behavioral interfaces.

Esper is an open-source Java-based system for CEP that provides a DSL for event processing called EPL. This DSL allows to formulate queries, called EPL statements, which continuously analyze events within a stream to detect situations of interest and produce a new stream of events containing properties selected from the matching events. Java objects, called *subscribers*, can then subscribe to this new event stream to be notified each time an event is inserted into the stream.

As we defined the event part of the ECA rules of our relationships as temporal patterns over either a stream of event occurrences or a stream of call notifications, CEP is particularly fitting to the realization of relationships. Moreover, as Esper is Java-based and open-source, it integrates well with our existing model execution framework.

#### Event manager

To streamline the integration of relationships into the architecture and avoid dependencies between behavioral interfaces, we define a component called the *event manager*. The event manager is implemented as an ECA rules engine configured by the active relationships. For each relationship, two streams are created: one for the environment-to-model direction and one for the model-to-environment direction. According to both the nature of its containing relationship (implementation or subtyping) and its direction (environment-to-model or model-to-environment), a stream contains either event occurrences or call notifications. Streams carrying event occurrences only accepts occurrences of events from the corresponding behavioral interface, based on its direction and on the nature of its containing relationship. The temporal patterns constituting the event part of accept and expose rules are registered to their corresponding stream, as defined by their relationship. The condition and action methods constituting the condition and action parts of the rules of the relationships are then hooked on their corresponding temporal pattern. Figure 10 illustrates the event manager, to which an implementation and a subtyping relationship have been registered.

At runtime, the event manager is responsible for dispatching event occurrences between relationships (that is, between their event streams). The event manager dispatches an event occurrence for translation to the event stream of a given relationship based on (1) the behavioral interfaces referenced by the relationship, (2) their supertype or subtype role in the relationship (for subtyping relationship only) and (3) the accepted or exposed nature of the event occurrences. Note that, if several registered relationships qualify for a given event occurrence, this occurrence is dispatched to each relationship.

For instance, the subtyping relationship between ActivatableInterface (the supertype) and ArduinoInterface (the subtype) shown in the upper part of Fig. 10 can receive occurrences of the activate and led_level_changed events, because activate is an accepted event from the supertype interface of the relationship, and led_level_changed is an exposed event from the subtype interface of the relationship. However, this relationship cannot receive occurrences of the activated event, as this is an exposed event from the supertype interface of the relationship: Such event occurrences can be emitted by the relationship but never received, as the relationship does not know how to handle occurrences of this event. For the same reason, this relationship cannot receive occurrences of the run event.

Additionally, the event manager is tasked with communicating with the other entities in the system. One such entity is the operational semantics of the xDSL, to which the event manager sends call requests and from which it receives call notifications. Precisely, how this is handled will be discussed in Sect. 5.2. Other possible entities are external tools sending accepted event occurrences to the event manager and/or being notified by the event manager of exposed event occurrences.

Note that, as it has been designed, this event manager is not specific to CEP-based relationships and can accommodate to any technology allowing relationships to offer the following two required services: (1) receiving event occurrences and (2) notifying of event occurrences (e.g., using runtime monitors). In fact, an envisioned approach to define relationships is to propose a dedicated, declarative event mapping language letting the language engineer define when and on what condition an event or sequence thereof should be mapped to another event or sequence thereof.

#### Modeling event occurrences in Esper

To use Esper, we need to map our event occurrences to event representations that can be processed by Esper. A range of possibilities are available, from Plain Java Objects (POJOs) to Maps to XML documents. We opted for modeling our events as POJOs, as we do not require the flexibility of Maps, and our implementation is exclusively Java-based, making XML both cumbersome and unnecessary. More specifically, we defined a wrapper class for EventOccurrence objects. This wrapper class declares two methods that are considered as event properties by the Esper runtime. The first method is the getEvent method, which returns the event of the occurrence. The second method is the getArgs method that takes an event parameter named as parameter and returns the value associated with that parameter. This allows Esper to access the different arguments of an event occurrence as a mapped property, by supplying the parameter name of the argument. For instance, the expression args(’someName’) returns the value provided for the event parameter named ’someName’.

As in our proposed strategy, call notifications are inserted into the event stream and manipulated by the Esper runtime, and we also need an Esper representation for them. Since call notifications are issued by the integration facade, which in our case is Java-based, the simplest solution for the proposed architecture is to model these call notifications as POJOs, as we do for event occurrences. Such POJOs point to the execution rule at the origin of the call notification, to a map associating the values supplied for each parameter of the execution rule in that particular call and for notifications of completed calls, to the value returned by the call.

#### Relationship design

With event occurrences and call notification made Esper-compatible, we can now look into the design process of implementation and subtyping relationships and their ECA rules, based on Esper and Java. We will then present a concrete example of the application of this process to our Arduino DSL running example.

*Design process* ECA rules are defined in the following manner. The event part of ECA rules is defined using EPL statements querying the stream corresponding to the nature of the rule (i.e., accept or expose). This allows to leverage the power of CEP to capture complex, potentially overlapping patterns of event occurrences. The condition and the action parts of a rule are written as Java methods to be called by the event manager when a complex event is detected by the EPL statement defined as the event part. The condition method takes complex events detected by the EPL statement as parameter and returns a Boolean value indicating whether the action method should be called or not. To be able to enforce domain-specific constraints, the condition method has access to the running model in addition to the triggering complex event to compute its result value. Conversely, the action method also takes as parameter the complex event that was detected by the EPL statement. The action method of accept rules returns either an array of event occurrences (for subtyping relationships) or an array of call requests (for implementation relationships), while the action method of expose rules always returns a single event occurrence. Access to the running model allows the action method to configure newly instantiated event occurrences (e.g., supplying event occurrences with parameters from the model).

*Concrete example* Figure 10 illustrates this design strategy by showing a more in-depth view of Fig. 9, which provides an overview of implementation and subtyping relationships between ActivatableInterface and ArduinoInterface. First, it highlights the fact that each relationship holds two event streams: a stream associated to accept ECA rules (next to labels **1** and **2**) and another associated to expose ECA rules (next to labels **3** and **4**).

Then, on the upper-left part of the figure (labeled **1**), the OnActivateButton accept rule of the subtyping relationship between ActivatableInterface and ArduinoInterface is detailed. The event stream observed by this rule contains ActivatableInterface accepted event occurrences. The event part of the OnActivateButton rule is an EPL statement that notifies its subscribers (i.e., the registered rules) whenever an occurrence of an event named activate is inserted on the event stream. When this happens, the subscribers receive a notification that carries the id parameter value, selected by the EPL statement through the args(’id’) expression. In the example, there are two subscribers, one of which (OnActivateSketch) is not shown. The other subscriber is the OnActivateButton rule. When notified, the evaluateCondition method, whose implementation is required of subscribers, is called. This method checks that the condition of the rule is satisfied. In the example, the implementation of this condition method performs a query on the running model, using the value provided by the complex event pattern of the event part of the rule. This is achieved using a utility method findElement which finds an element of the provided class with the provided name (here buttonId) in the provided model (here the running model). Then, if the condition is satisfied, the subscriber performs the action of the rule by calling its execute method, which translates the triggering event occurrence into two new event occurrences. This is effectively done by instantiating the new occurrences (using dedicated utility methods in our example) and returning them in an array to be inserted in the correct event stream (in this case, the stream of ArduinoInterface event occurrences).

On the lower-left part of the figure (labeled **2**), the design of the accept rules of the ArduinoInterface implementation relationship is detailed. It is very similar to that of the subtyping relationship, the event stream containing ArduinoInterface accepted event occurrences instead of ActivatableInterface ones. The accepted rules observing this event stream instantiate and return call requests for specific execution rules. The OnButtonPressed rule shown in the figure detects occurrences of the button_pressed event and converts them directly (as no condition is specified) into call requests for the PushButton.press execution rule of the operational semantics.

Then, on the lower-right part of the figure (labeled **3**) is detailed the design of the expose rule of the implementation relationship. The event stream associated with this rule contains the call notifications issued by the operational semantics. The OnSetLed rule detects call notifications for the SetLed.execute execution rule on the event stream and converts them into occurrences of the led_level_changed event, to which it supplies the referred led and its new level.

Finally, on the upper-right part of the figure (labeled **4**) is detailed the OnLEDOffOn expose rule of the subtyping relationship. This rule observes an event stream containing ArduinoInterface exposed event occurrences. The EPL statement constituting the event part of the rule specifies that it is triggered whenever a succession of two led_level_changed event occurrences with alternating level parameter values but identical led parameter values is observed in a sliding window of two events. The action part of the rule translates the triggering complex event into an occurrence of the activated event with the id of the LED as a parameter value.

### Metalanguage integration

In addition to providing a unified way to define the accepted and exposed events for any xDSL, our approach aims to be agnostic of the metalanguage used to define the operational semantics of an xDSL. This means that the behavioral interface language and the design of the event manager and relationships must work for any xDSL, regardless of the metalanguage used to define its operational semantics.

To achieve this, an integration facade for the event manager must be defined. This facade is tasked with translating call requests into actual behavior and behavior into call notifications, thereby bridging the gap between the event manager (and the implementation relationships therein) and the operational semantics.

In this section, we propose such an integration facade to enable the approach for xDSLs whose operational semantics is defined using an object-oriented metalanguage such as Java and orchestrated by an execution engine. Note that the proposed integration facade is intended for sequential model execution. Adapting the approach to concurrent model execution only requires to define an appropriate integration facade. First, we present what must be provided by this execution engine, which is considered as a prerequisite for the proposed facade. Next, we detail the inner workings of the integration facade. Finally, we show how this facade is interfaced with the aforementioned execution engine.

#### Execution engine

The proposed approach considers that a preexisting *execution engine* applies the operational semantics of the considered xDSL on the running model. Such an execution engine must be able to notify external components when it starts or stops and when it applies execution rules that alter the model state. More precisely, the engine only sends notifications for execution rules annotated as a *stepping rule*, which are executions rule producing an observable execution step when applied. Regarding the Arduino DSL presented in Sect. 2, only stepping rules are presented.

The state of the model is considered observable and alterable at the time notifications are made and handled; hence, the possible observable states reached during an execution are heavily dependent on the granularity of the declared stepping rules in a semantics. This notification mechanism can not only be used to attach interactive debuggers [6] and trace constructors [7] to the execution. We explain later how we leverage this notification mechanism to enable exposed events and run-to-completion call requests. The design of an execution engine is described in more detail in our previous work [5, 6] and can be summarized as the following operations:

- start does the ensuing actions:
  - load the considered xDSL;
  - load the model to be executed;
  - register the execution observers;
  - prepare the initial model state;
  - set the running attribute of the engine to true;
  - notify registered observers that it is starting.
- stop sets the running attribute to false and notifies execution observers that the engine is stopping.
- callExecutionRule starts the application of a specific execution rule of the operational semantics. If it is a stepping rule, the engine notifies observers at the beginning and at the end of the execution of the rule. Note that depending on the metalanguage, an execution rule may trigger the nested execution of other stepping rules, in which case observers are also notified when the nested execution of these stepping rules begins or ends. For instance, in the Arduino model shown in Fig. 2, calls to SetLed.execute will be nested within calls to If.execute, which will in turn be nested within calls to Sketch.run. Note that no distinction is made between the notifications from nested and non-nested rule calls.
- registerObserver registers a component as an *observer* that gets notified when the execution of a stepping rule begins or ends and when the engine starts or stops. When an observer gets registered, an associated *priority policy* needs to be supplied as well. Such a policy provides, for each kind of notification, the priority at which the observer must be notified. This operation is called by the execution engine during the initialization phase, to register a predefined set of execution observer, retrieved from a configuration file for instance, but it can also be called at any time.

The specification of this component is by design as generic as possible to be able to cover a wide range of metalanguages. As such, it provides an abstraction over the multiple execution engines dedicated to various metalanguages available in the GEMOC Studio (see Sect. 5.3). The implementation of this component is, however, heavily dependent on the metalanguage used to implement the operational semantics, especially regarding the procedure to dynamically call an arbitrary execution rule (e.g., using java.lang.reflect.Method.invoke if the semantics is implemented in Java).

Using these operations, a user (e.g., a modeler, a tool) is able to execute a model by starting the engine and then demanding the execution of one or several execution rules of the semantics (e.g., a *run* method responsible for the complete execution). In the following subsections, we explain how the integration facade can also use these operations for managing call requests and notifications.

#### Overview of the metalanguage integration facade

To bridge the gap between implementation relationships and the execution engine, we define an integration facade concentrating on the following two activities: (1) waiting for execution rule call requests from implementation relationships and performing the requested calls and (2) issue execution rule call notifications to implementation relationships.

To be able to perform these activities, the integration facade has two requirements that need to be fulfilled. First, it needs a mechanism to wait for execution rule call requests to arrive. To that effect, our approach relies on a *blocking queue* to store the call requests received from implementation relationships. Call requests can be retrieved from the queue using the poll and the take operations, which behave differently when the queue is empty: take suspends the execution and waits for an element to be available, while poll simply returns null. Second, the integration facade needs to be able to call execution rules defined as part of the operational semantics of an xDSL. This task is delegated to the execution engine and its callExecutionRule operation.

With these requirements fulfilled, an execution with the proposed integration facade unfolds as follows:

- The integration facade is notified of the start of the execution by the execution engine. It enters its execution rule call scheduling loop: The execution is repeatedly suspended when the call request queue is empty and resumed when call requests are queued.
- Implementation relationships send call requests to the integration facade, which are added to the call request queue.
- The engine informs the integration facade when it is safe to process the queued call requests, i.e., when starting or ending stepping rule calls. In such cases, the integration facade first checks whether a run-to-completion call request is currently being executed. If that is the case, the call request queue is left untouched. Otherwise, the queued call requests are sequentially delegated to the execution engine.
- The integration facade is notified that a stepping rule call is about to start or has ended and forwards this notification to implementation relationships.

#### Metalanguage integration facade operations

We hereby present how the integration facade achieves these different tasks through a set of operations.
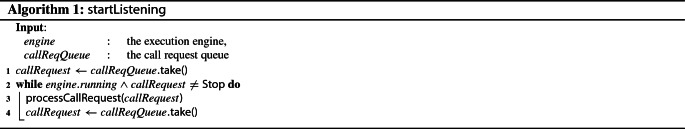


startListening*and*stopListening. These internal operations are used to start and stop the call request handling loop. Algorithm 1 shows startListening. As long as the execution engine is running, the first call request of the call request queue (lines 1–2 and 4 of Algorithm 1) is retrieved. When the take operation is called on the queue, the execution is suspended if the queue is empty—which only happens if no execution rule is currently executing—and resumes as soon as a request is added. Finally, the call request is processed using the processCallRequest operation (line 3 of Algorithm 1). The stopListening operation consists of inserting an instance of a special Stop call request into the call request queue (mechanism know as a *poison pill* [14]), thereby stopping the call request handling loop.

queueCallRequest. This operation is called by the event manager to insert a request to call the provided execution rule with the provided arguments into the call request queue. Note that, at the start of the execution, no actual execution takes place until a first call request is queued. For instance, in the case of the Arduino DSL, the execution only starts once a run event occurrence is received: This event occurrence enqueues, through a call to the queueCallRequest operation, a request for a call to Sketch.run on the sketch parameter of that event occurrence. This call request is then processed, which starts the execution.
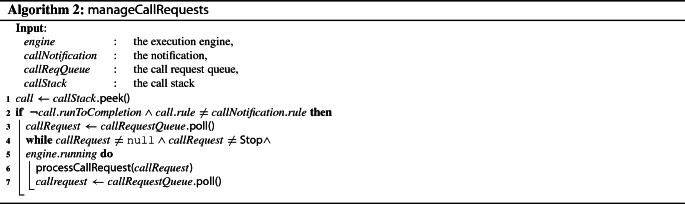


manageCallRequests This internal operation is similar to startListening, except that it does not suspend the execution when the queue of call requests is empty. It is called when the integration facade is notified that the running model is in a consistent state, and thus, that pending call requests can be safely handled. As explained previously, this is the case before and after the execution of stepping rules. Algorithm 2 shows the behavior of this operation. When it is called, the integration facade first checks that the currently executed call request did not ask for run-to-completion behavior. For this, the call request on top of the stack is inspected (line 1) and two conditions are checked: if it should not be treated as run-to-completion and if its associated execution rule is different from the stepping rule that triggered the notification (line 2). The first condition prevents the processing of a call request, while a run-to-completion call request is being handled. The second condition prevents the processing of additional call requests before the processing of the current one gets to start, which would otherwise happen when the rule associated with the current call request is a stepping rule. If both conditions allow it, the non-blocking poll operation is used to iterate over all call requests in the queue and process them using the processCallRequest operation (lines 3–7), exiting the loop if the engine stops or if the Stop call request is encountered. Otherwise, the call request queue is left untouched, to be processed at a later time, as the operation returns immediately.
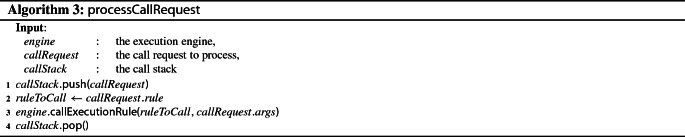


processCallRequest This internal operation, detailed in Algorithm 3, is used to process a single execution rule call request. First, the call request is pushed on a *call stack* (line 1). Then, the execution rule to call is retrieved from the call request (line 2), and the call is delegated to the execution engine (line 3). Once this call returns, the call request is popped from the call stack (line 4). This call stack keeps track of the call requests that are currently being handled and is used to enforce the potential run-to-completion nature of call requests by preventing the handling of other call requests while a run-to-completion one is being executed.

#### Integration with the execution engine

During its initialization phase, the execution engine instantiates and registers the integration facade as an observer from a configuration file. In the following, we detail how the integration facade reacts to the different notifications sent by the execution engine, combining the presented operations to achieve proper event handling.

- *notifyStart*: The call request handling loop is started, using the startListening operation.
- *beforeStep*: The manageCallRequests operation is called to process the call request queue, given that the call request currently under execution (if any) is not a run-to-completion call request.
- *afterStep*: call notifications are forwarded to implementation relationships, which decide if they should result in an exposed event occurrence. The facade then behaves as for *beforeStep* notifications.
- *notifyStop*: the stopListening operation is called to halt the call request handling loop.

In the event that all execution rule calls issued from the startListening operation terminate without a *notifyStop* notification being received, the call request handling loop suspends the execution, waiting for either a Stop request or a call request to be queued and thus instantly processed. Note that, when the integration facade is registered as an observer of the execution, an accompanying priority policy is supplied, specifying that it receives *notifyStart* and *afterStep* notifications last, but receives *beforeStep* notifications first. This allows the facade to work with other potential execution observers. For instance, a trace constructor needs to receive *beforeStep* notifications after the integration facade: Otherwise, it would record the start of an execution step when in fact another step could be triggered, given that there is a pending call request in the queue of the integration facade.

### Tool support

We implemented our approach as part of the GEMOC Studio [5], a language and modeling workbench atop the Eclipse platform [25]. The metamodel of the behavioral interface language is defined using Ecore, and the event manager is written in both Java and Xtend. The source code is available on Github.^3^

The language workbench of the GEMOC Studio offers multiple metaprogramming approaches to define the operational semantics of a DSL (e.g., Java/Kermeta [20], xMOF [24] or Henshin [2]), as well as one execution engine for each approach. Our implementation of the event manager is agnostic to the kind of execution engine that is used, in accordance with Sect. 5, and comes with a metalanguage integration facade for the Java/Kermeta-based execution engine.

In order to make use of the approach, a reflective event injection GUI was designed, allowing to send and receive event occurrences from running models and complementing the existing generic omniscient debugger for xDSLs defined in the language workbench [6]. We detail this tool in the next section.

### Language engineering scenarios

In this subsection, we describe several language engineering scenarios and the role played by our approach in their realization.

*Specific tooling development* Language engineers can leverage the definition of behavioral interfaces to develop tooling that is specific to one or more interfaces, such as a domain-specific graphical view of the event occurrences sent to and received from the model. Such tooling can then be used with any model conforming to a DSL implementing the supported interfaces.

*Generic tooling development* Language engineers can leverage the reflexive access provided by the behavioral interface metalanguage to develop generic interaction-centric tooling. At runtime, from the definition of the xDSL to which the running model conforms, such tools can retrieve the list of behavioral interface implemented (directly or transitively) by the DSL. Then, from these behavioral interfaces, generic tools can discover the events whose occurrences can be accepted or exposed by the running model, along with their parameters. Language engineers can then implement generic tooling revolving around events and their occurrences.

*Tooling for multi-model interaction* Going further than the previous scenario, language engineers can define tools that handle interaction with concurrently running models conforming to different DSLs. Both broadcasting event occurrences to eligible models and sending event occurrences to a single model can be supported. Conversely, language engineers can define tools to receive event occurrences from one or all eligible running models. This is very close to generic tooling development: The main difference is that instead of gathering the implemented behavioral interfaces from one running model, the tool lists the implemented behavioral interfaces from all running models. From there, the language engineer has all the information required to implement the desired event sending behavior. Alternatively, this capability can also be implemented for interface-specific tools, for a predefined set of behavioral interfaces.

*Model coordination* This scenario requires a complementary approach such as B-COoL [22] to actually coordinate models through occurrences of events from their respective behavioral interfaces. With such a complementary approach, modelers are able to leverage the behavioral interface defined for the involved xDSLs to define how a specific set of models conforming to these DSLs are coordinated.

## Evaluation

In this section, we first evaluate whether the proposed approach fulfills each of its requirements, which are listed in Sect. 2.2; then, we conclude by summarizing and discussing the results, as well as the threats to validity.

### Interface definition and implementation (Req. 1)

To evaluate how well the proposed approach fulfills **Req. 1**, we apply the approach on two existing xDSLs to enable interaction with their conforming models. In a first time, we apply the approach on the Arduino DSL presented in Sect. 2, a very specific DSL. In a second time, we apply the approach to a subset of UML State Machines in conformance with the Precise Semantics of UML State Machines (PSSM) specification [33], which is a general and standardized modeling language. We then report on the process.

*Executable DSL I: Arduino* The first xDSL on which we apply the approach is the Arduino DSL presented in Sect. 2. The behavioral interface directly implemented by the Arduino DSL has been introduced in Fig. 6 and contains three accepted events (run, button_pressed and button_released) and one exposed event (led_level_changed). The implementation relationship defined between this behavioral interface and the operational semantics of the Arduino DSL is straightforward:

- run occurrences are translated into call requests to the Sketch.run execution rule,
- button_pressed and button_released occurrences are translated into call requests to the Button.press and Button.release execution rules, and
- calls to the SetLed.execute execution rule are translated into led_level_changed occurrences, with the new level being directly queried from the model.

In total, the implementation relationship itself required, for each ECA rule, around 5–6 lines of Java code for the method bodies, while we were able to define a library specific to Esper-based implementation relationships that can be reused for any implementation relationship. The definition of this library required 94 lines of Java code.

*Executable DSL II: UML state machines* The second xDSL on which we applied the approach is a subset of UML State Machines in conformance with the Precise Semantics of UML State Machines (PSSM) specification [33]. Since we focused on reproducing the event-related behavior of UML State Machines with our approach, we implemented a relevant subset of the language defined as follows:

- The implementation supports initial, final, entry point, exit point, fork, join and terminate pseudo-states. History, choice and junction pseudo-states are not supported as they take no part in the event-handling behavior of UML State Machines.
- Although PSSM is an extension of fUML [31], which gives semantics for UML activity diagrams, our implementation of PSSM only covers UML State Machines.
- State machine redefinition is not supported since this is not related to the event handling logic.

Among the execution rules of the operational semantics, four rules stand out: The StateMachine.run starts the execution of the model, StateMachine.signalReceived notifies a StateMachine that it received a SignalOccurrence, StateMachine.callPerformed notifies a StateMachine that call was performed, and Behavior.execute launches the execution of a Behavior.

**Fig. 11:**
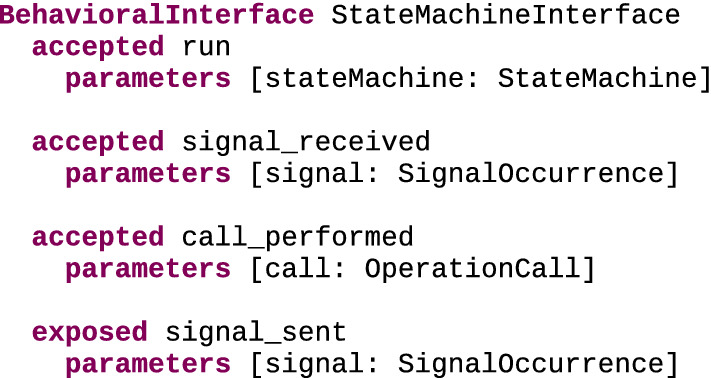
Behavioral interface for UML state machines

Figure 11 shows the StateMachineInterface we defined for UML State Machines. This interface contains three accepted events that are described thereafter. The run event triggers the initialization required to start the execution of the state machine. The signal_received event takes a signal occurrence as parameter and triggers run-to-completion steps. As signals potentially contain parameters, signal occurrences can provide values for these parameters. The call_performed event takes an operation call as parameter and also triggers run-to-completion steps. The interface also contains one exposed event: signal_sent, which takes a signal as parameter. Note that this event normally occurs in the activity diagrams used to define the behavior of states and transitions of UML State Machines, not in the state machines themselves. However, as our implementation does not include activity diagrams, instead using stubs thereof, we added the signal_sent event to the StateMachineInterface.

As for the Arduino DSL, the implementation relationship defined between the StateMachineInterface and UML State Machines is straightforward. First, run, signal_received and call_performed occurrences are translated into call requests for the run, signalReceived and callPerformed execution rules, with a one-to-one mapping between event occurrence arguments and execution rule arguments. Second, call notifications for the Behavior.execute execution rule are translated into signal_sent occurrences.

In total, the implementation relationship itself required, for each ECA rule, around 5–8 lines of Java code for the method bodies, and we were able to reuse the same library for Esper-based implementation relationships that we defined when applying the approach to the Arduino DSL.

*Fulfilling****Req. 1***. We successfully applied the approach on two xDSLs, one very specific and the other more general. In the process, we defined a library that language engineers can reuse to define their Esper-based implementation relationships. This allowed to keep the amount of lines of code required to implement each ECA rules very low, at around 5 to 8 lines of Java code.

This shows that the proposed metalanguage is expressive enough to define in a unified way the possible interactions with models conforming to these two DSLs. The soundness of the enabled model interactions is up to the language engineer, as it depends on the execution semantics of the DSL. The language engineer has full control over which behavior can be triggered by event occurrences, and the conditions of the ECA rules allow the language engineer to perform extensive checks before accepting or emitting event occurrences. Therefore, the approach fulfills **Req. 1** for the two considered DSLs.

### Realizing reflective tools (Req. 2)

We evaluate **Req. 2** by demonstrating how the proposed approach provides genericity through reflection. In more details, we demonstrate how the reflection capabilities provided by our metalanguage for behavioral interfaces enables the development of tools compatible with any xDSL implementing a behavioral interface. Consequently, language engineers applying the approach to define a behavioral interface and an implementation relationship for their xDSLs are able to provide some degree of interactive tool support for free, as their DSLs directly benefit from reflective tool support. We first demonstrate how a test runner able to run test suites for any xDSL can be defined. We then show that the approach enables the definition of a GUI to configure and send accepted event occurrences, and receive exposed event occurrences in accordance with the definition of their events. Combined together, these two tools allow for practical definition and execution of test cases, for instance, for non-regression testing. Indeed, the GUI can be used to configure accepted event occurrences, send them and store both these occurrences and the ones received in return under the form of a test scenario that can then be run by the test runner.

*Reflective tool I: test runner* As a first reflective tool, we implemented a test runner which is able to process a previously defined test suite to drive the execution of a model under test and check an oracle.

Test cases are defined in a test suite point to an xDSL definition, as well as a model under test conforming to that DSL. Each test case also contains both a test scenario as a sequence of event occurrences to send to the model and an oracle as event occurrences that must be received, interleaved with the test scenario. The test runner, implemented as a launch configuration for the GEMOC Studio, reads provided test suites and iterates over the test cases they contain. For each test case, the test runner starts a new execution using the operations detailed in Sect. 5.2.1. Then, the test runner alternates sending event occurrences from the test scenario of the test case and waiting for event occurrences from the oracle of the test case. The implementation of this tool is possible due to the unified representation of events and their occurrences provided by the approach, as Event and EventOccurrence elements. Indeed, this both enables to define test suites that contain event occurrences from any behavioral interface and allows the test runner to send and receive event occurrences while staying agnostic to their behavioral interface.

Using this tool, we were able to check the conformance of our implementation of a subset of UML State Machines with the Precise Semantics for UML State Machines (PSSM). We retrieved the available test suite designed for Papyrus and, using a model transformation, converted it into a test suite model compatible with our test runner. We also used the test runner to execute test cases on Arduino models. The general pattern followed by these tests is as follows: The test runner sends an accepted event occurrences, awaits for one or more resulting exposed event occurrences and repeats this until the test case is finished.

*Reflective tool II: event injection GUI.* As a second reflective tool, we implemented a reflective event injection GUI for the GEMOC Studio that leverages the active behavioral interfaces to (1) allow the user to create and send accepted event occurrences to the running model and (2) listen to exposed event occurrences and display them in a log. In more details, the tool features a list of all implemented and supertype interfaces. For each behavioral interface selected in this list, the tool provides an event occurrence configurator per accepted event defined in the interfaces. By reflectively analyzing the defined parameters for each accepted event, the GUI is able to provide well-suited controls to configure an occurrence of these events, such as a text field letting users enter the value of their choosing for parameters whose type is a string (e.g., the id parameter of the activate event). Alternatively, the configurator for button_pressed event occurrences provides a list of all model elements whose type matches the parameter type (PushButton in this case), as well as a browse button that lets users select a predefined model element in an arbitrary resource located in the workspace. Finally, the GUI provides a log of exposed event occurrences listing all the received event occurrences.

As we implemented our approach and tools within the GEMOC Studio, we are able to use the reflective event injection GUI in conjugation with the generic debugger already provided by the GEMOC Studio [6]. Using this extended debugger, we are able to pause the execution, queue event occurrences, use stepping operators (forward and backward), define breakpoints and resume a paused execution to evaluate the impact of queued event occurrences on this execution. This can be used on any model conforming to any DSL developed with any of the metalanguages provided by the language workbench for which an integration facade is defined.

*Fulfilling****Req. 2*** No tool-specific line of code is required to interact with running models, indicating that the event manager component provides a sufficiently expressive API for both tools. In addition, by design, the event manager guarantees that event handling does not result in undefined behavior, as it forbids simultaneous calls to execution rules. The condition part of ECA rules also guarantees that execution rules are only called in execution states allowed by the language engineer.

Therefore, by implementing these two tools and showing how they can be used with both UML State Machines and Arduino DSL, we showed that reflective tools can be built that leverage behavioral interfaces to both discover how to interact with a running model and do so in a sound and unified way. This means that the approach fulfills **Req. 2** for these two tools.

### Interface subtyping (Req. 3)

We show how the approach fulfills **Req. 3** by combining implementation and subtyping relationships defined over an xDSL and its behavioral interfaces to define an event abstraction hierarchy. This is to define interactive tools tailored for a given behavioral interface (i.e., a given set of top-level events), yet that can be reused across all xDSLs implementing this interface, either directly or transitively. In more details, we define a subtyping relationship between the ActivatableInterface interface shown in Fig. 7 and both the ArduinoInterface and the StateMachineInterface. We then discuss how this enables the interchangeability of the two considered xDSLs, and what the reaped benefits are.

*Subtyping with Arduino DSL* As the subtyping relationship between ActivatableInterface and ArduinoInterface has already been presented in Figs. 9 and 10 , we will summarize its content thereafter.

This subtyping relationship features two accept rules translating activate events into either a run event occurrence, or a sequence of two event occurrences: a bu-tton_pressed occurrence followed by a button_relea-sed occurrence. This depends on whether the id parameter value of the event occurrence refers to a Sketch or to a PushButton. The relationship also features one expose rule translating led_on and led_off occurrences into activated occurrences. This is a more complex ECA rule as its event part consists of a pattern that matches sequences of led_off occurrences followed by led_on occurrences on a specific time frame. Only when a match is found, the involved event occurrences can be translated into a activated occurrence.

As for implementation relationships, we were able to define a library specific to Esper-based subtyping relationships that we reused for all subtyping relationships we defined. By leveraging this library, the ECA rules of the subtyping relationship required around 3 to 5 lines of Java code per method body.

**Fig. 12:**
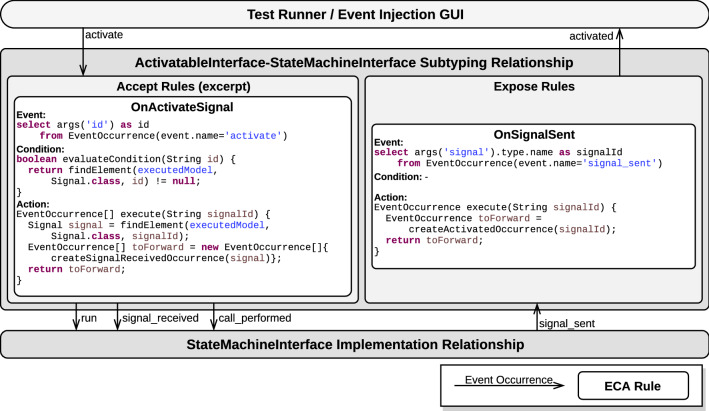
Subtyping relationship between ActivatableInterface and StateMachineInterface

*Subtyping with UML state machines* Figure 12 details the content of the OnActivateSignal and OnSignalSent ECA rules of the subtyping relationship between ActivatableInterface (as a supertype) and StateMachineInterface (as a subtype).

The left part of the figure highlights the fact that activate occurrences (from ActivatableInterface) are translated into run, signal_received and call_performed occurrences (from StateMachineInterface). The OnActivateSignal rule is detailed. Upon detecting an activate occurrence, it first checks that a Signal element with a name identical to the value supplied for the id parameter of the activate occurrence exists in the executed model. If that is the case, the rule translates the original event occurrence into a signal_received occurrence from StateMachineInterface. This new occurrence is configured to carry a newly instantiated occurrence of the proper signal.

The right part of the figure highlights the fact that signal_sent occurrences (from StateMachineInterface) are translated into activated occurrences (from ActivatableInterface) through the OnSignalSent rule. This rule is straightforward as it maps occurrences of the signal_sent event to occurrence of the activated event carrying the name of the signal (i.e., the type of the signal occurrence carried out by signal_sent event occurrences) as the value of their id parameter.

In total, the ECA rules of the subtyping relationship each required around 3–7 lines of Java code for the method bodies, as we were able to reuse the library for Esper-based subtyping relationships.

*Fulfilling****Req. 3*** In this demonstration case, we defined ActivatableInterface as a common supertype of both the Arduino DSL and UML State Machines through subtyping relationships between ActivatableInterface and both ArduinoInterface and StateMachineInterface. In the process, we defined a library dedicated to Esper-based subtyping relationships, allowing us to keep the number of lines of Java code required to define each ECA rule of these relationship between 3 and 7 per method body.

Once defined, the subtyping relationships allow the previously defined reflective tools to be indiscriminately used with models conforming to either DSL, sending and receiving event occurrences from the ActivatableInterface interface in both cases. For example, the test runner can be used to check whether an Arduino model being a realization of a State Machine model behaves in the same expected way, by running the exact same test suite on both models, provided this test suite is designed with event occurrences from the ActivatableInterface interface. We were thus able to capitalize upon this and use tools with events from ActivatableInterface with models conforming to the Arduino DSL and with models conforming to UML State Machines, thereby fulfilling **Req. 3** for these two DSLs.

### Summarized results

In summary, the proposed metalanguage allowed us to define explicit behavioral interfaces for xDSLs that specify how modelers and tools can soundly interact with running models.

Second, having such explicit behavioral interfaces, combined to the explicit API of the event manager allows the development of reflective tools reproducing essential features available in common executable modeling tools. This allows tools to be generic through reflection, as was demonstrated by using two such tools (a test runner and an event injection GUI) with two different xDSLs.

Finally, explicit behavioral interfaces allow subtyping relationships to be defined between them, which can in turn enable substitutability of xDSLs and thus genericity through abstraction. This then allows to define tools that are specific to a given behavioral interface but can in fact be used by any xDSL having this interface as a supertype. It also allows to substitute an xDSL by another one tailored for the task at hand (e.g., analysis of state machines versus simulation plus code generation targeting Arduino platforms).

### Threats to validity

*Internal validity* As we are experienced in using the GEMOC Studio, we might have overlooked limitations to our approach that would make it hard to use for language engineers. Conducting a user study to assess the usefulness of our tools and the usability of our approach is an important direction of our future work.

*External validity* We verified that our approach yields its benefits for two xDSLs and two generic tools, which externally threatens the ability of our approach to be generalized to multiple DSLs and tools. However, the two selected xDSLs are relevant and representative of the languages supported by the approach as their abstract syntax is defined as a metamodel and their execution semantics is defined as a discrete-event operational semantics written in an object-oriented language and orchestrated by an execution engine. This indicates that the approach could be generalized to these languages, given that their operational semantics provides the necessary granularity to enable the proper handling of events through calls to existing execution rules. In the opposite case, a refactoring of the operational semantics in accordance with the good practice of the separation of concerns is required.

Another threat to validity is that the DSLs used in the evaluation were implemented with interaction in mind and thus presented the appropriate execution rules to correctly design their implementation relationships. This externally threatens the ability of our approach to be applied to any existing language without modifying it. However, the intent of our contribution is to provide a new way of designing DSLs and is thus geared toward the definition of new languages or the (possibly substantial) refactoring of existing ones, not toward opportunistic reuse of existing languages. As this is also an interesting potential application of the approach, we consider it as a future work direction.

### Critical discussion

While the approach works well for xDSLs whose conforming models are similar to state/transition systems, it presents some limitations when working with xDSLs that have time-related concepts, such as the Arduino DSL. More precisely, defining an ECA rule similar to the activate rule for PushButton elements that allows for a customized duration between pressing and releasing a button (and more generally between two event occurrences) would require to be able to specify waiting times before specific event occurrences are sent by the event manager. Since the approach works at the language level, the issue is then to decide on a waiting time that will fit all conforming models, or to find a way to derive or define this waiting time on a model-by-model basis. Additionally, expressing time durations also requires a time unit, which could either be a generic unit (e.g., execution steps) or a domain-specific one (e.g., number of turns for a camshaft), specified at the language or model level, or even a real-time one such as seconds or milliseconds.

User-wise, the adoption of the approach by language engineers has an impact on how they work, and most notably on the way they design the execution semantics of their xDSL. Indeed, to enable the definition of events at any level of granularity, execution rules must be designed for a single task, and internal behavior needs to be clearly separated from potentially external behavior. This means that, when applying the approach on an existing DSL, some refactoring might be necessary to be able to define meaningful events. However, we believe that these requirements fit the good practice of the separation of concerns, advocating for methods to be dedicated to one precise task. Therefore, as long as language engineers implemented the execution semantics of their xDSL according to the separation of concern, little to no refactoring is necessary for adopting the approach.

Finally, the use of the Stop event discussed in Sect. 5.2.3 and the existence of a run event in both ArduinoInterface and StateMachineInterface indicate that a kind of “system” behavioral interface, dedicated to execution specific events (e.g., starting and stopping the execution, pausing it, waiting) would be beneficial. This in turn hints at another purpose for behavioral interfaces, defined at the metalanguage level, which is worth investigating.

## Related work

While a sizable amount of work has been done on language interfaces, most of it is dedicated to structural language interfaces, i.e., language interfaces for interacting with models on the structural level. Such works include the Meta Object Facility [32], the Object Constraint Language [34], the Language Server Protocol (LSP)^4^ [37] and all the work on model typing [11, 15, 39]. Our contribution instead stands on the behavioral side of language interfaces. Yet, as it leverages work on model typing for event parameters, it builds on structural language interfaces. It can also complement them, e.g., pairing behavioral interface with LSP to provide both remote editing and interactive support for models. Nevertheless, in this section we precisely scope the considered related works to those dedicated to interacting with models on the behavioral level, which we divide in two categories: those working at the model level and those working at the language level.

### Model-level

Yakindu^5^ is a state-of-the-art tool providing support for the definition of interaction interfaces for Statecharts models, which can be seen as behavioral model interfaces: They specify input and output events for a model and generate code allowing to send corresponding event occurrences to the model and listen to the emitted event occurrences. In comparison, our approach works for any language within our scope as it is used at the language level, thereby allowing interaction with any conforming model.

An emerging standard to provide a behavioral interface in the area of simulation is the Functional Mockup Interface (FMI).^6^ Simulation models are converted into executables called Functional Mockup Units (FMUs) which implement the standardized FMI, and each is accompanied with an XML model description of the interfaces of the unit. FMUs are mostly used for continuous models where time steps are performed, variables are set with initial values, and some variables may be observed during execution. This makes FMIs a kind of behavioral model interface for continuous models while, in contrast, our approach is situated at the language level and takes an event-driven perspective.

### Language-level

In [22], the authors advocate for the need of language behavioral interface for coordinating the execution of heterogeneous models and define one such interface as an extension of the abstract syntax of an FSM language. In [10], the author similarly mentions behavioral language interfaces as a means to coordinate the execution of models conforming to heterogeneous languages. The focus of this work is, however, not on behavioral language interfaces themselves and uses a specific kind of behavioral interface, defined for a specific purpose: model coordination. The interface used in this work is similar to the most precise interface we detail in Sect. 4.1.3. In [21], Kindler presents the Event Coordination Notation (ECNO) which allows to model event coordination for object-oriented languages. All those approaches can be seen as complementary to our work, as they either rely on or could work with behavioral language interfaces to achieve model coordination.

In [43], the authors report on the use of Event-B at the language level to enable formally verified behavioral interaction with conforming models. With the methodology described in the paper, Event-B is used as a metalanguage to define the translational semantics of an industrial DSL. This means that, provided our approach was extended to support translational semantics, this work could be complementary to our approach. In [35], the authors introduce a methodology to define a transformation from the metamodel of a DSL to Concurrent Object-Oriented Petri-Nets (CO-OPN), thereby providing a translational semantics for xDSLs. From the obtained CO-OPN specification, a prototype can be generated, in turn enabling the simulation of conforming models. In [18], the authors present a methodology leveraging DSL embedding in Scala to provide several execution semantics for a given xDSL, in a way that is transparent to the language user, through the use of a language interface implemented by these execution semantics. The authors also propose to leverage the internal nature of DSLs defined through their methodology to compose their semantics together. As these works offer metaprogramming approaches for defining the execution semantics of xDSLs, they are complementary to our approach. Indeed, provided a respective metalanguage integration facade is defined for them, these approaches could be connected to ours, enabling the implementation of behavioral interface and direct reuse of generic, interaction-centric tools.

In some of our previous work [9], we advocate for the inclusion of an event definition metamodel in the specification of xDSLs, to which occurrences of domain-specific events conform. Our proposed behavioral interface metalanguage is a more expressive mean to define such an event definition metamodel. Beyond this extended expressivity, our proposed metalanguage relies on behavioral typing to enable implementation and subtyping relationships, and its execution semantics allows to safely interact with running models. In [27], Meyers et al. presented the ProMoBox approach, which includes the generation of an *input metamodel* from a DSL definition, which can also be seen as a behavioral interface. However, this interface is only used for model checking, while our contribution is geared toward model execution. In [26], the closest to our work, an approach to augment an xDSL with reactive capabilities and generate a corresponding domain-specific *test language* is presented. This requires to enrich the abstract syntax with event-related concepts and to accommodate for placeholder rules in the operational semantics, to be later replaced by calls to the test engine, which manages test cases and events. In contrast, our approach does not require such a rewriting as the management of events is done implicitly before the start and at the end of the execution of stepping rules, nor does it require to alter the abstract syntax, thus facilitating reuse of legacy xDSLs. Additionally, in our approach, behavioral interfaces are used to define behavioral types for xDSLs through nominal typing and subtyping, which enables the provision of tools that can be generic (through reflection) or specific to families of xDSLs (through abstraction).

To summarize, there is an existing work focusing on bringing interaction capabilities on a model-by-model basis for specific languages or for continuous models. At the same time, several works point to the need for language-level behavioral interfaces and rely on such interfaces defined in an ad hoc way. While there is existing work providing, at the language level, the means to interact with conforming models in a way that is related to our approach, it is done more intrusively on xDSLs, and interaction is only enabled with a dedicated test engine and does not allow the definition of additional (generic or domain-specific) tools. Finally, several works are complementary to our proposed approach as they explore the use of event-centric languages as metalanguages to define the execution semantics of executable DSLs, which could then be mapped to behavioral interfaces through integration facades and implementation relationships.

## Conclusion and future work

Interacting with running models is crucial for many tasks, ranging from automated testing to communication between heterogeneous models. To address this problem, we proposed an approach to attribute a behavioral type to xDSLs under the form of explicit behavioral interfaces declaring the accepted and exposed events that can be used to communicate with conforming models. This enables the definition of generic tools for xDSLs through reflection. We complemented this by providing an approach to define subtyping relationships between behavioral interfaces. This in turn enables substitutability of xDSLs sharing a common supertype, thus enabling the definition of generic tools though abstraction. We provide semantics for behavioral interfaces implementation and subtyping in the form of an event manager that acts as an intermediary between the external tools and the running model. We implemented our approach for the GEMOC Studio, a language and modeling workbench for xDSLs. We demonstrated the value of our approach through two demonstration cases based on the implementation of two generic event-centric tools and their usage on two xDSLs through their respective implemented interface in a first time, and through their common supertype interface in a second time.

Perspectives for future work can be sorted in two categories: extending/improving the approach and leveraging the approach. Future work extending the approach includes leveraging formally defined behavior for xDSLs and behavioral interface to automatically infer the implementation and subtyping relationships between them. Such an extension would take inspiration from existing work (e.g., [41]) on model refinement operators that are behavior-preserving and could leverage existing languages for CEP with a formally defined semantics such as [1]. Exploring how behavioral interfaces can be leveraged at the metalanguage level is also an interesting line of research. Another perspective for future work is to investigate whether a model-level configuration or refinement of language-level behavioral interfaces makes sense and allows to solve problems such as the definition of model-specific waiting times between event occurrences. In a somewhat opposite direction, another perspective is determining the relevance of adjoining a behavioral specification (e.g., a labeled transition system) to behavioral interfaces that can serve as a language-level protocol to both provide the expected semantics from implementing DSLs and detect and deal with issues such as out-of-order event occurrences, the challenge being that all conforming models then need to comply to this protocol. Another envisioned extension is to identify the needs of concurrent execution semantics to design the best-suited integration facade for such execution semantics, allowing to best leverage concurrency. Also conducting a user study is included where a group of participants take on the role of language engineers and are asked to define a couple of behavioral interfaces and the relationships between them, in order to evaluate and improve the usability of the approach. Another prospect for future work is conducting a thorough performance and scalability analysis reporting on the overhead induced by handling multiple relationships during the same execution. A last perspective in this category is categorizing the different kinds of legacy execution semantics and identifying the challenges to address to define implementation relationships on top of them.

Perspectives for leveraging the approach include extending generic support for V&V activities on xDSLs (e.g., debugging, testing), such as the definition of generic test coverage metrics for test case generation for xDSLs, or identifying various purposes for which behavioral interfaces can be designed (e.g., debugging interface, animation interface, execution management interface, ...). Also extending capabilities are included for generic runtime monitoring for models conforming to xDSLs, to be used during both debugging (e.g., for defining conditional breakpoints) and testing (e.g., to serve as test oracles). Another perspective is to explore how the proposed approach can be integrated in a context of model coordination, either to simultaneously test or debug models, or to coordinate the execution of models representing different parts of a system. An additional extension of the approach is to redefine classic design patterns for languages. For instance, providing a definition of the adapter pattern for xDSLs would allow to define mappings between behavioral interfaces that do not support a subtyping relationship (e.g., when an $n{:}\,m$ mapping between events would be required). A final perspective is the definition of a generic execution engine able to execute models conforming to DSLs whose operational semantics is defined across several metalanguages, through the use of multiple integration facades contributed by each metalanguage used to define the operational semantics. These last two perspectives would both contribute to the line of research on the composition of DSLs [19].

## Footnotes

1 https://www.eclipse.org/papyrus/.

2 https://github.com/tetrabox/examples-behavioral-interface.

3 https://github.com/eclipse/gemoc-studio.

4 http://langserver.org.

5 https://www.itemis.com/en/yakindu/state-machine/.

6 http://fmi-standard.org.
